# Poly[diaquatris(μ_6_-4,6-dioxo-1,4,5,6-tetra­hydro-1,3,5-triazine-2-carboxylato)tripotassium]

**DOI:** 10.1107/S1600536814007569

**Published:** 2014-04-09

**Authors:** Sarra Soudani, Emmanuel Aubert, Emmanuel Wenger, Christian Jelsch, Isabelle Gautier-Luneau, Cherif Ben Nasr

**Affiliations:** a Laboratoire de Chimie des Matériaux, Faculté des sciences de Bizerte, 7021 Zarzouna, Tunisie; b Cristallographie, Résonance Magnétique et Modélisations (CRM2), UMR CNRS 7036, Institut Jean Barriol, Université de Lorraine, BP 70239, Bd des Aiguillettes, 54506 Vandoeuvre-les-Nancy, France; c Université Joseph Fourier, Institut Néel, CNRS, Département MCMF, 25 rue des Martyrs, 39042 Grenoble cedex 9, France

## Abstract

The asymmetric unit of the title compound, [K_3_(C_4_H_2_N_3_O_4_)_3_(H_2_O)_2_]_*n*_, contains two potassium cations (one in general position, one located on a twofold rotation axis), one and a half oxonate anions (the other half generated by twofold symmetry) and one water mol­ecule. As a result of the twofold symmetry, one H atom of the symmetric anion is statistically occupied. Both potassium cations are surrounded by eight oxygen atoms in the form of distorted polyhedra. Adjacent cations are inter­connected by oxygen bridges, generating layers parallel to (100). The aromatic ring system of the oxonate anions link these layers into a network structure. The crystal packing is stabilized by N—H⋯O, O—H⋯O and O—H⋯N hydrogen bonds, three of which are bifurcated. In addition, inter­molecular π–π stacking inter­actions exist between neighboring aromatic rings with a centroid–centroid distance of 3.241 (2) Å.

## Related literature   

For applications of metal-organic coordination materials, see: Yaghi *et al.* (2003 ▶); Janiak (2003 ▶); Lalart *et al.* (1981 ▶); Mori *et al.* (2005 ▶, 2006 ▶); Dybtsev *et al.* (2004 ▶). For studies and properties of oxonic acid, see: Lalart *et al.* (1981 ▶); Pancheva (1977 ▶); Cihak *et al.* (1968 ▶). For comparable inter­atomic distances in related structures, see: Sheldrick & Poonia (1986 ▶); Cuesta *et al.* (2003 ▶); Pike (1976 ▶). For π–π stacking inter­actions, see: Janiak (2000 ▶). For a multipolar atom model transfered from the ELMAM2 electron density database, see: Domagała *et al.* (2012 ▶). For fractal analysis of the residual electron density, see: Meindl & Henn (2008 ▶).
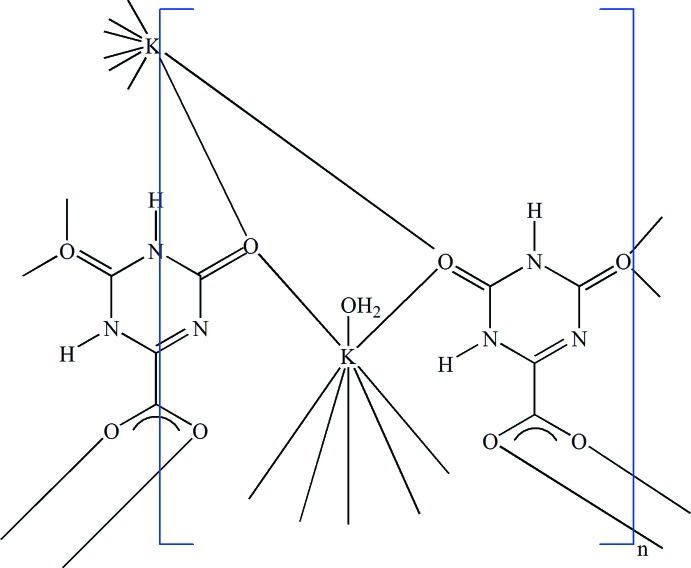


## Experimental   

### 

#### Crystal data  

[K_3_(C_4_H_2_N_3_O_4_)_3_(H_2_O)_2_]
*M* *_r_* = 621.55
Monoclinic, 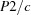

*a* = 7.0284 (2) Å
*b* = 7.6736 (2) Å
*c* = 19.2668 (4) Å
β = 99.355 (2)°
*V* = 1025.30 (5) Å^3^
*Z* = 2
Mo *K*α radiation
μ = 0.77 mm^−1^
*T* = 110 K
0.16 × 0.13 × 0.07 mm

#### Data collection  

Bruker APEXII CCD diffractometer
Absorption correction: multi-scan (*SADABS*; Bruker, 2012 ▶) *T*_min_ = 0.887, *T*_max_ = 0.948
34196 measured reflections
2953 independent reflections
2637 reflections with > 2.0σ(*I*)
*R*_int_ = 0.038

#### Refinement  

*R*[*F* ^2^ > 2σ(*F* ^2^)] = 0.027
*wR*(*F* ^2^) = 0.067
*S* = 0.93
2953 reflections
190 parameters
14 restraints
Only H-atom coordinates refined
Δρ_max_ = 0.50 e Å^−3^
Δρ_min_ = −0.36 e Å^−3^

### 

Data collection: *APEX2* (Bruker, 2012 ▶); cell refinement: *SAINT* (Bruker, 2012 ▶); data reduction: *SAINT*; program(s) used to solve structure: *MoPro* (Jelsch *et al.*, 2005 ▶); program(s) used to refine structure: *MoPro*; molecular graphics: *ORTEP-3 for Windows* (Farrugia, 2012 ▶); software used to prepare material for publication: *MoPro*.

## Supplementary Material

Crystal structure: contains datablock(s) global, I. DOI: 10.1107/S1600536814007569/wm5013sup1.cif

Structure factors: contains datablock(s) I. DOI: 10.1107/S1600536814007569/wm5013Isup2.hkl

CCDC reference: 995461

Additional supporting information:  crystallographic information; 3D view; checkCIF report

## Figures and Tables

**Table 1:** Hydrogen-bond geometry (Å, °)

*D*—H⋯*A*	*D*—H	H⋯*A*	*D*⋯*A*	*D*—H⋯*A*
N3_3—H3_3⋯O9_3	1.02 (1)	2.20 (1)	2.6086 (7)	102 (1)
N5_3—H5_3⋯O8_3^i^	1.02 (1)	1.93 (1)	2.9070 (7)	162 (1)
N5_3—H5_3⋯O9_3^i^	1.02 (1)	2.56 (1)	3.3977 (6)	140 (1)
N12_4—H12_4⋯O18_5	1.016 (5)	1.984 (8)	2.9628 (7)	160.9 (4)
N12_4—H12_4⋯O18_5^ii^	1.016 (5)	2.659 (9)	3.1515 (8)	110 (2)
N14_4—H14_4⋯O16_4^iii^	1.03 (1)	2.23 (1)	3.1553 (6)	150 (2)
N14_4—H14_4⋯O16_4^iv^	1.03 (1)	2.23 (1)	3.1553 (7)	150 (2)
O18_5—H18*B*_5⋯O16_4^v^	0.96 (1)	2.58 (1)	3.3013 (8)	133 (2)
O18_5—H18*A*_5⋯N1_3^i^	0.97 (1)	1.93 (1)	2.8927 (9)	173 (1)

Symmetry codes: (i) 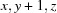; (ii) 
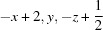
; (iii) 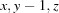; (iv) 
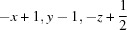
; (v) 
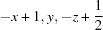
.

**Fig. 1.**
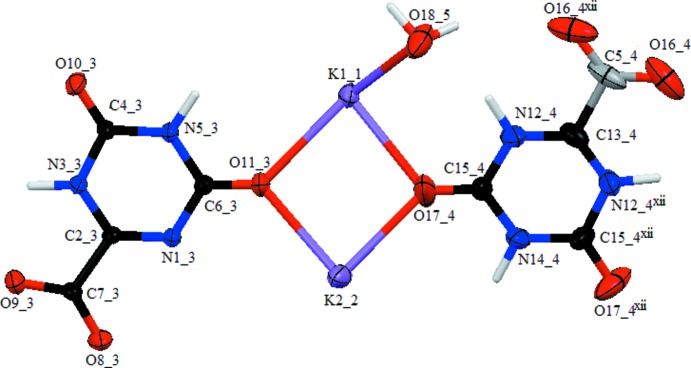
The basic structure units in the structure of (I), showing 50% probability displacement ellipsoids and spheres of arbitrary radius for the H atoms. [Symmetry code: (xii) -x + 1, y, -z + 1/2.]

**Fig. 2.**
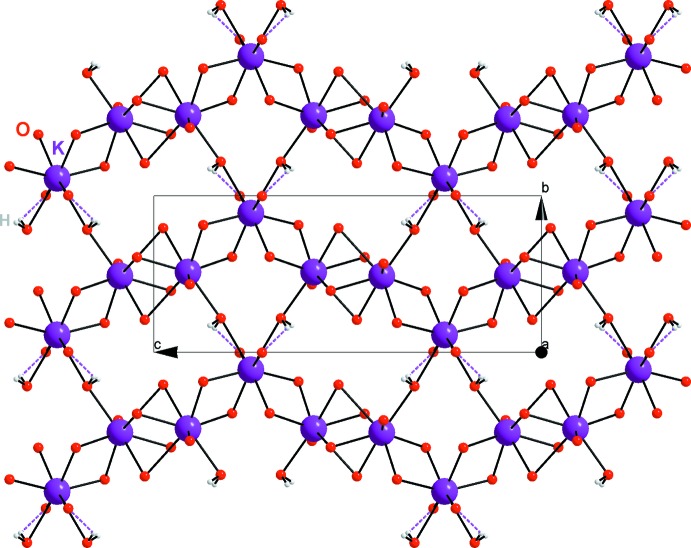
Projection of a layer in the crystal structure of (I) along the a-axis. Hydrogen bonds are shown as broken lines.

**Fig. 3.**
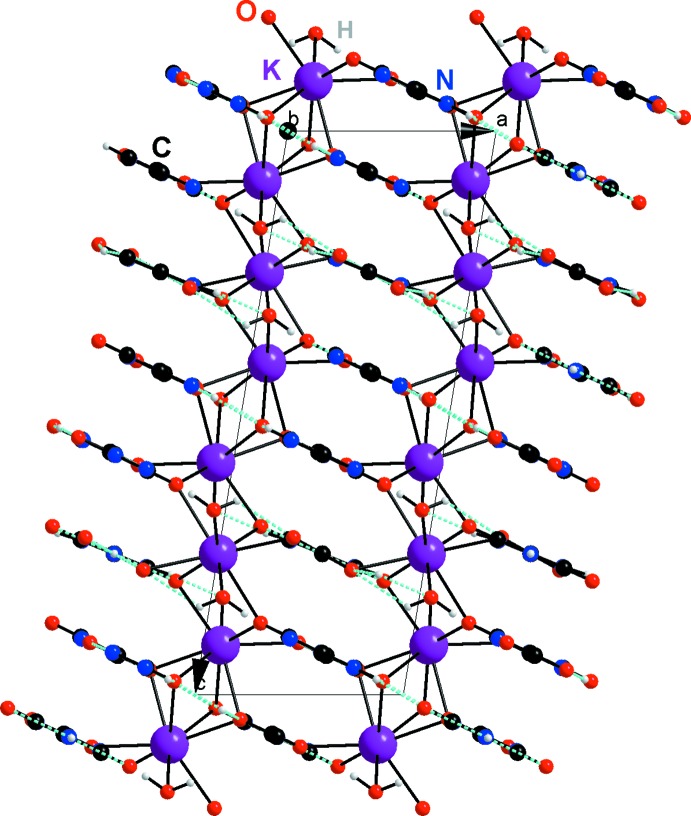
Projection of the crystal structure of (I) along the b-axis. Hydrogen bonds are shown as broken lines.

**Fig. 4.**
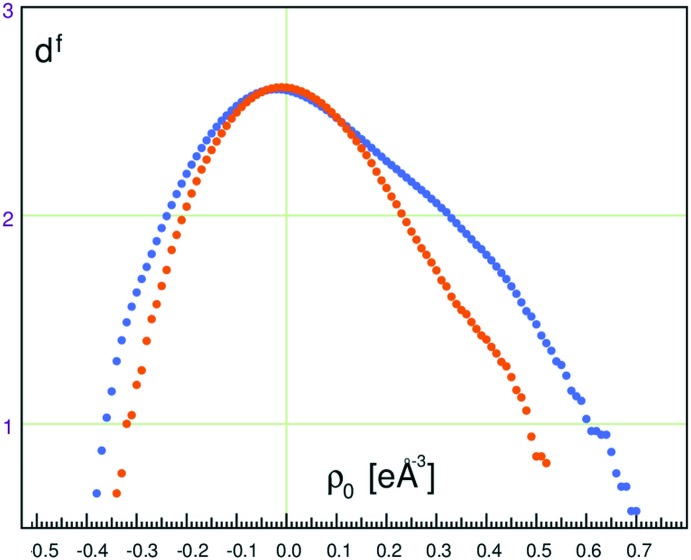
Fractal analysis of the Fourier residual electron density. Blue: spherical atom model; orange: transferred multipolar atom model.

## supplementary crystallographic information

### 1. Comment

Oxonic acid has antibacterial and antiviral properties (Pancheva, 1977); it is a competitive inhibitor of pyrimidine biosynthesis (Cihak *et al.*, 1968) and occupies an unique biologic position by being the only effective precursor in the biosynthesis. Besides being biologically important, oxonic acid has also been of interest in coordination and supramolecular chemistry. Despite its importance in biochemistry, physical chemistry studies of oxonic acid are rare, probably due to its low solubility and, particularly, to the instability of oxonic acid solutions which easily decarboxylate into 5-azauracil. The study of the kinetics of metal-oxonic acid decarboxylation has been conducted some time ago (Lalart *et al.*, 1981).

In recent years, much attention has been paid for crystal engineering of metal-organic coordination compounds (Yaghi *et al.*, 2003). This arises not only from fundamental properties of these materials, such as their intriguing topological frameworks, but also from their unexpected potential applications in various fields such as engineering, device manufacturing or materials science (Janiak, 2003; Mori *et al.*, 2005, 2006; Dybtsev *et al.*, 2004).

As a contribution to the investigation of the above materials, we report here the crystal structure of the hydrated potassium salt of oxonic acid, K_3_(C_4_H_2_N_3_O_4_)_3_^.^2H_2_O, (I).

The asymmetric unit of the structure of (I) contains two potassium cations (one in general position, one located on a twofold rotation axis), one water molecule and one and a half molecules of the oxonic acid anion (1,4,5,6-tetrahydro-4,6-dioxo-1,3,5-triazine-2-carboxylate), the second half completed by a twofold rotation axis (Fig. 1). Due to symmetry, one hydrogen atom (H12_4) of this anion is equally disordered between two equivalent sites. The two potassium cations are octa-coordinated to oxygen atoms in the form of distorted cubic antiprisms. The coordination environment of K1_1 is defined by three oxygen atoms of carboxylate groups, four oxygen atoms of carbonyl groups and one oxygen atom of the water molecule. K2_2 is surrounded by four oxygen atoms of carbonyl groups, two oxygen atoms of carboxyl groups and two oxygen atoms of water molecules. Each K1_1 potassium atom shares four bridging oxygen atoms (O9_3^v^, O9_3^vi^, O10_3^iii^ and O10_3^iv^) with a symmetry-related cation K1_1^i^, and two bridging oxygen atoms (O11_3 and O17_4) with the potassium cation K2_2 (for symmetry codes, see Table). The K—O distances, ranging from 2.6893 (6) to 3.1649 (6) Å are similar than in related potassium complexes (Sheldrick & Poonia, 1986).

The K1_1—K1_1^i^ and K1_1—K2_2 distances are 3.7662 (3) and 4.2236 (3) Å respectively, also in good agreement with related structures (Cuesta *et al.*, 2003). The potassium cations are connected by oxygen bridges to form layers parallel to (100) (Fig. 2). Between two adjacent layers, located at *x*≈ 0, are inserted the aromatic rings and are linked through N—H···O and O—H···N hydrogen bonds into a three-dimensional network (Fig. 3). Among these hydrogen bonds, three are bifurcated: N14_4—H14_4···(O16_4i, O16_4iii), N12_4—H12_4···(O18_5, O18_5v) and N5_3—H5_3···(O8_3ii, O9_3ii) (details and symmetry codes in Table 1).

In the organic anion the N—C distances spread between 1.297 (2) and 1.392 (2) Å, clearly indicating π-electron delocalization over the C_3_N_3_ ring. The shortest N1_3-C2_3 distance, involving the deprotonated nitrogen atom, has the strongest double bond character. The N12_4—C13_4 bond involving the half-protonated nitrogen atom has an intermediary length of 1.320 (2) Å compared to N1—C2 (1.297 (2) Å) and N3—C2 (1.356 (2) Å). All in all, the interatomic distances and the bond angles have their usual values (Pike, 1976).

In addition, intermolecular π···π stacking interactions exist between neighboring aromatic rings with a centroid-to-centroid distance of 3.241 (2) Å, which is less than 3.8 Å, the maximum value regarded as relevant for such stacking interactions (Janiak, 2000).

### 2. Experimental

Potassium oxonate (4,6-dihydroxy-1,3,5-triazine-2-carboxylic acid potassium salt) was obtained as a commercially available salt (Aldrich, 97%) and was dissolved in a minimum amount of water at 323 K. The solution was slowly cooled in two days in an incubator from 323 K to 277 K. Crystals of the title compound could then be isolated after two days and were subjected to X-ray diffraction analysis.

### 3. Refinement

After initial refinement with *SHELXL97*, the structure was further refined with the program *MoPro* (Jelsch *et al.*, 2005) using a multipolar atom model transfered from the ELMAM2 electron density database (Domagała *et al.*, 2012). The *R*(*F*) factor improved from 4.3 to 3.4%. The residual difference electron density showed a positive/negative peak when the nitrogen atom N12_4 was modeled as deprotonated or fully protonated, respectively. Due to the twofold symmetry of this anion the hydrogen atom H12_4 was modelled with half-occupancy on the two crystallographically equivalent sites. The other H atom positions were refined using distance restraints; the target values were 1.01 (2) and 0.97 (2) Å for N—H and O—H bond lengths, respectively. In the oxonate moieties, angle similarity restraints (σ = 0.2°) were also applied to the C—N—H triplets. The H atoms were restrained to remain close to the planes of the oxonate moieties (σ = 0.03). The H atoms of the water molecule were refined using two O—H distance and one distance similarity restraints, and the target of the H—O—H angle was set to 105.0 (2)°. The fractal analysis of the residual electron density (Meindl & Henn, 2008) in Fig. 4 shows a more symmetric curve for the multipolar model, with notably a reduced shoulder on the positive side.

### Crystal data

**Table:**

[K_3_(C_4_H_2_N_3_O_4_)_3_(H_2_O)_2_]	*Z* = 4
*M**_r_* = 310.77	*F*(000) = 628
Monoclinic, *P*2/*c*	*D*_x_ = 2.013 Mg m^−^^3^
Hall symbol: -P 2yc	Mo *K*α radiation, λ = 0.71073 Å
*a* = 7.0284 (2) Å	θ = 2.7–31.0°
*b* = 7.6736 (2) Å	µ = 0.77 mm^−^^1^
*c* = 19.2668 (4) Å	*T* = 110 K
β = 99.355 (2)°	Prism, colourless
*V* = 1025.30 (5) Å^3^	0.16 × 0.13 × 0.07 mm

### Data collection

**Table:**

Bruker APEXII CCD diffractometer	2953 independent reflections
Radiation source: fine-focus sealed tube	2637 reflections with > 2.0σ(*I*)
Mirror monochromator	*R*_int_ = 0.038
ω scans	θ_max_ = 30.4°, θ_min_ = 2.7°
Absorption correction: multi-scan (*SADABS*; Bruker, 2012)	*h* = −9→9
*T*_min_ = 0.887, *T*_max_ = 0.948	*k* = 0→10
34196 measured reflections	*l* = 0→27

### Refinement

**Table:**

Refinement on *F*^2^	Primary atom site location: structure-invariant direct methods
Least-squares matrix: full	Secondary atom site location: difference Fourier map
*R*[*F*^2^ > 2σ(*F*^2^)] = 0.027	Hydrogen site location: difference Fourier map
*wR*(*F*^2^) = 0.067	Only H-atom coordinates refined
*S* = 0.93	*w* = 1/[σ^2^(*F*_o_^2^) + (0.04*P*)^2^ + 0.5*P*] where *P* = (*F*_o_^2^ + 2*F*_c_^2^)/3
2953 reflections	(Δ/σ)_max_ = 0.005
190 parameters	Δρ_max_ = 0.50 e Å^−^^3^
14 restraints	Δρ_min_ = −0.36 e Å^−^^3^

### Special details

**Table:**

Refinement. Refinement of *F*^2^ against reflections. The threshold expression of *F*^2^ > σ(*F*^2^) is used for calculating *R*-factors(gt) and is not relevant to the choice of reflections for refinement. *R*-factors based on *F*^2^ are statistically about twice as large as those based on *F*, and *R*-factors based on ALL data will be even larger.

### Fractional atomic coordinates and isotropic or equivalent isotropic displacement parameters (Å^2^)

**Table:**

	*x*	*y*	*z*	*U*_iso_*/*U*_eq_	Occ. (<1)
K1_1	1.08353 (4)	0.48766 (4)	0.412084 (14)	0.01245 (4)	
K2_2	1	0.11136 (5)	0.25000	0.01989 (6)	
O8_3	1.45467 (13)	−0.43420 (12)	0.40758 (5)	0.01477 (13)	
O9_3	1.76362 (13)	−0.39318 (12)	0.45621 (5)	0.01479 (13)	
O10_3	1.88936 (13)	0.20722 (12)	0.47622 (5)	0.01638 (13)	
O11_3	1.25768 (13)	0.18741 (12)	0.37279 (5)	0.01419 (13)	
N1_3	1.41234 (15)	−0.07072 (14)	0.40074 (5)	0.00943 (13)	
N3_3	1.73874 (15)	−0.05408 (13)	0.45147 (5)	0.00987 (14)	
N5_3	1.57096 (15)	0.20278 (14)	0.42495 (5)	0.00984 (14)	
C2_3	1.57561 (17)	−0.14319 (16)	0.42608 (6)	0.00913 (16)	
C4_3	1.74401 (17)	0.12642 (15)	0.45228 (6)	0.01027 (16)	
C6_3	1.40543 (17)	0.11050 (16)	0.39764 (6)	0.00935 (15)	
C7_3	1.59851 (17)	−0.34380 (16)	0.43032 (6)	0.00942 (16)	
H3_3	1.8583 (19)	−0.121 (2)	0.4729 (8)	0.01180*	
H5_3	1.559 (3)	0.3346 (13)	0.4231 (9)	0.01177*	
N12_4	0.66429 (16)	0.65016 (15)	0.27330 (6)	0.01528 (15)	
N14_4	0.50000	0.3866 (2)	0.25000	0.0163 (2)	
C13_4	0.50000	0.7302 (2)	0.25000	0.0151 (3)	
C15_4	0.6706 (2)	0.46992 (18)	0.27535 (7)	0.01688 (19)	
C5_4	0.50000	0.9310 (3)	0.25000	0.0207 (3)	
O16_4	0.34766 (19)	1.00230 (15)	0.22125 (6)	0.0303 (2)	
O17_4	0.81757 (16)	0.38998 (15)	0.29846 (6)	0.03083 (18)	
H14_4	0.50000	0.253 (2)	0.25000	0.01920*	
H12_4	0.7874 (15)	0.7204 (7)	0.287 (2)	0.01819*	0.50
O18_5	1.05599 (15)	0.78304 (15)	0.32766 (6)	0.02606 (16)	
H18A_5	1.173 (2)	0.828 (3)	0.3556 (10)	0.03898*	
H18B_5	0.953 (2)	0.845 (3)	0.3439 (10)	0.03898*	

### Atomic displacement parameters (Å^2^)

**Table:**

	*U*^11^	*U*^22^	*U*^33^	*U*^12^	*U*^13^	*U*^23^
K1_1	0.00912 (12)	0.01238 (13)	0.01537 (13)	0.00076 (9)	0.00058 (9)	0.00085 (9)
K2_2	0.0193 (2)	0.01123 (18)	0.0248 (2)	0	−0.00922 (16)	0
O8_3	0.0112 (4)	0.0094 (4)	0.0229 (5)	−0.0023 (3)	0.0001 (3)	−0.0009 (4)
O9_3	0.0117 (4)	0.0102 (4)	0.0209 (4)	0.0021 (3)	−0.0020 (3)	0.0011 (3)
O10_3	0.0091 (4)	0.0102 (4)	0.0273 (5)	−0.0019 (3)	−0.0046 (3)	0.0019 (4)
O11_3	0.0112 (4)	0.0110 (4)	0.0184 (4)	0.0029 (3)	−0.0034 (3)	−0.0010 (3)
N1_3	0.0074 (4)	0.0077 (4)	0.0124 (4)	0.0005 (4)	−0.0009 (3)	0.0000 (4)
N3_3	0.0073 (4)	0.0069 (4)	0.0145 (5)	−0.0002 (3)	−0.0012 (4)	0.0007 (4)
N5_3	0.0078 (4)	0.0076 (5)	0.0131 (4)	0.0005 (4)	−0.0014 (4)	0.0007 (4)
C2_3	0.0076 (5)	0.0069 (5)	0.0122 (5)	−0.0010 (4)	−0.0004 (4)	−0.0007 (4)
C4_3	0.0079 (5)	0.0065 (5)	0.0155 (5)	0.0002 (4)	−0.0008 (4)	0.0008 (4)
C6_3	0.0078 (5)	0.0087 (5)	0.0109 (5)	−0.0001 (4)	−0.0005 (4)	−0.0006 (4)
C7_3	0.0086 (5)	0.0062 (5)	0.0132 (5)	−0.0002 (4)	0.0013 (4)	−0.0002 (4)
N12_4	0.0117 (5)	0.0110 (5)	0.0231 (5)	−0.0020 (4)	0.0024 (4)	−0.0021 (4)
N14_4	0.0195 (8)	0.0079 (7)	0.0186 (7)	0	−0.0051 (6)	0
C13_4	0.0180 (9)	0.0093 (8)	0.0198 (8)	0	0.0090 (7)	0
C15_4	0.0156 (6)	0.0120 (6)	0.0200 (6)	0.0032 (5)	−0.0062 (5)	−0.0028 (5)
C5_4	0.0336 (11)	0.0083 (8)	0.0244 (9)	0	0.0178 (8)	0
O16_4	0.0455 (7)	0.0169 (5)	0.0337 (6)	0.0130 (5)	0.0218 (5)	0.0081 (4)
O17_4	0.0267 (6)	0.0269 (6)	0.0325 (6)	0.0161 (5)	−0.0144 (5)	−0.0108 (5)
O18_5	0.0161 (5)	0.0236 (5)	0.0345 (6)	−0.0058 (4)	−0.0076 (4)	0.0098 (4)

### Geometric parameters (Å, º)

**Table:**

K1_1—K1_1^i^	3.7662 (3)	N1_3—C2_3	1.297 (2)
K1_1—K2_2^ii^	4.2236 (3)	N3_3—C4_3	1.386 (2)
K1_1—O17_4	2.7419 (9)	N3_3—C2_3	1.356 (2)
K1_1—O11_3	2.7714 (7)	N3_3—H3_3	1.015 (19)
K1_1—O18_5	2.7782 (9)	N5_3—C4_3	1.375 (2)
K1_1—O10_3^iii^	2.9272 (7)	N5_3—C6_3	1.390 (2)
K1_1—O10_3^iv^	3.1649 (6)	N5_3—H5_3	1.015 (19)
K1_1—O9_3^v^	2.6893 (6)	C2_3—C7_3	1.549 (2)
K1_1—O9_3^vi^	2.6893 (6)	N12_4—C15_4	1.384 (2)
K2_2—O17_4	2.7342 (9)	N12_4—C13_4	1.320 (2)
K2_2—O11_3	2.7972 (7)	N12_4—H12_4	1.016 (18)
K2_2—O16_4^vii^	2.7236 (9)	N14_4—C15_4	1.376 (2)
K1_1—O8_3^viii^	2.6916 (6)	N14_4—C15_4^xii^	1.376 (2)
K2_2—O18_5^ix^	2.9240 (6)	N14_4—H14_4	1.03 (3)
K2_2—O18_5^x^	2.9240 (6)	C13_4—N12_4^xii^	1.320 (2)
K2_2—O17_4^ii^	2.7342 (9)	C13_4—C5_4	1.541 (3)
K2_2—O11_3^ii^	2.7972 (6)	C15_4—O17_4	1.222 (3)
O8_3—C7_3	1.246 (2)	C5_4—O16_4	1.249 (2)
O9_3—C7_3	1.245 (2)	C5_4—O16_4^xii^	1.249 (2)
O10_3—K1_1^xi^	2.9272 (7)	O16_4—K2_2^xiii^	2.7236 (9)
O10_3—C4_3	1.220 (2)	O17_4—K1_1	2.7419 (9)
O11_3—K1_1	2.7714 (7)	O17_4—K2_2	2.7342 (9)
O11_3—K2_2	2.7972 (7)	O18_5—K1_1	2.7782 (9)
O11_3—C6_3	1.222 (2)	O18_5—H18B_5	0.96 (3)
N1_3—C6_3	1.392 (2)	O18_5—H18A_5	0.97 (3)
			
O17_4—K1_1—O11_3	80.13 (3)	C6_3—N5_3—H5_3	116 (4)
O17_4—K1_1—O18_5	77.41 (3)	C15_4—N12_4—C13_4	119.8 (2)
O17_4—K1_1—O10_3^iii^	80.33 (3)	C15_4—N12_4—H12_4	120 (1)
O11_3—K1_1—O18_5	120.67 (3)	C13_4—N12_4—H12_4	120 (1)
O11_3—K1_1—O10_3^iii^	76.18 (4)	C15_4—N14_4—C15_4^xii^	124.6 (5)
O18_5—K1_1—O10_3^iii^	148.7 (2)	C15_4—N14_4—H14_4	117.7 (2)
O17_4—K2_2—O11_3	79.81 (4)	C15_4^xii^—N14_4—H14_4	117.7 (2)
O17_4—K2_2—O16_4^vii^	71.63 (4)	N12_4^xii^—C13_4—C5_4	117.7 (4)
O11_3—K2_2—O16_4^vii^	111.78 (4)	O17_4—C15_4—N14_4	122.2 (5)
K1_1^xi^—O10_3—C4_3	129.30 (5)	O17_4—C15_4—N12_4	122.2 (5)
C6_3—N1_3—C2_3	117.8 (4)	O16_4—C5_4—O16_4^xii^	128.0 (6)
C4_3—N3_3—C2_3	121.8 (4)	O16_4—C5_4—C13_4	115.98 (11)
C4_3—N3_3—H3_3	118.9 (9)	O16_4^xii^—C5_4—C13_4	116.0 (5)
C2_3—N3_3—H3_3	119.2 (9)	K2_2^xiii^—O16_4—C5_4	141.0 (2)
C4_3—N5_3—C6_3	124.2 (4)	H18B_5—O18_5—H18A_5	105 (5)
C4_3—N5_3—H5_3	120 (4)		
			
K1_1—O17_4—K2_2—O11_3	5.20 (14)	N1_3—C6_3—N5_3—C4_3	2.8 (3)
K1_1—O17_4—C15_4—N14_4	−141.9 (5)	N1_3—C6_3—N5_3—H5_3	−178.2 (7)
K1_1—O17_4—C15_4—N12_4	37.7 (3)	N1_3—C2_3—N3_3—C4_3	−0.1 (3)
K1_1—O11_3—K2_2—O17_4	−5.11 (14)	N1_3—C2_3—N3_3—H3_3	177 (2)
K1_1—O11_3—C6_3—N5_3	−38.9 (3)	N3_3—C4_3—N5_3—C6_3	−1.1 (3)
K1_1—O11_3—C6_3—N1_3	139.8 (5)	N3_3—C4_3—N5_3—H5_3	180 (2)
K2_2—O17_4—K1_1—O11_3	−5.25 (14)	N3_3—C2_3—N1_3—C6_3	1.8 (3)
K2_2—O17_4—K1_1—O18_5	119.46 (17)	N5_3—C4_3—N3_3—C2_3	−0.3 (3)
K2_2—O17_4—C15_4—N14_4	47.3 (3)	N5_3—C4_3—N3_3—H3_3	−177 (2)
K2_2—O17_4—C15_4—N12_4	−133.1 (5)	N5_3—C6_3—N1_3—C2_3	−3.1 (3)
K2_2—O11_3—K1_1—O17_4	5.09 (14)	C4_3—N3_3—C2_3—C7_3	−179.5 (3)
K2_2—O11_3—K1_1—O18_5	−63.78 (12)	C6_3—O11_3—K1_1—O17_4	−177.7 (3)
K2_2—O11_3—C6_3—N5_3	137.5 (4)	C6_3—O11_3—K1_1—O18_5	113.4 (4)
K2_2—O11_3—C6_3—N1_3	−43.7 (3)	C6_3—O11_3—K2_2—O17_4	177.5 (3)
O8_3—C7_3—C2_3—N3_3	−179.6 (2)	C6_3—N1_3—C2_3—C7_3	−178.8 (2)
O8_3—C7_3—C2_3—N1_3	0.9 (3)	C7_3—C2_3—N3_3—H3_3	−3 (4)
O9_3—C7_3—C2_3—N3_3	−0.2 (4)	N12_4—C15_4—N14_4—H14_4	179.05 (3)
O9_3—C7_3—C2_3—N1_3	−179.7 (4)	N12_4—C13_4—C5_4—O16_4	173.1 (3)
O10_3—C4_3—N5_3—C6_3	179.966 (4)	N14_4—C15_4—N12_4—C13_4	1.9 (4)
O10_3—C4_3—N5_3—H5_3	1 (4)	N14_4—C15_4—N12_4—H12_4	−174 (3)
O10_3—C4_3—N3_3—C2_3	178.64 (8)	C13_4—N12_4—C15_4—O17_4	−177.7 (3)
O10_3—C4_3—N3_3—H3_3	2 (4)	C15_4—O17_4—K1_1—O18_5	−53.7 (5)
O11_3—K1_1—O17_4—C15_4	−178.4 (3)	C15_4—N12_4—C13_4—C5_4	178.97 (10)
O11_3—K1_1—O18_5—H18B_5	159.5 (3)	C5_4—C13_4—N12_4—H12_4	−5.2 (7)
O11_3—K1_1—O18_5—H18A_5	−95 (4)	O17_4—K1_1—O18_5—H18B_5	89 (3)
O11_3—K2_2—O17_4—C15_4	177.5 (3)	O17_4—K1_1—O18_5—H18A_5	−166 (2)
O11_3—C6_3—N5_3—C4_3	−178.4 (2)	O17_4—C15_4—N14_4—H14_4	−1.3 (3)
O11_3—C6_3—N5_3—H5_3	1 (4)	O17_4—C15_4—N12_4—H12_4	6.4 (8)
O11_3—C6_3—N1_3—C2_3	178.2 (3)		

Symmetry codes: (i) −*x*+2, −*y*+1, −*z*+1; (ii) −*x*+2, *y*, −*z*+1/2; (iii) *x*−1, *y*, *z*; (iv) −*x*+3, −*y*+1, −*z*+1; (v) −*x*+3, −*y*, −*z*+1; (vi) *x*−1, *y*+1, *z*; (vii) −*x*+1, *y*−1, −*z*+1/2; (viii) *x*, *y*+1, *z*; (ix) *x*, *y*−1, *z*; (x) −*x*+2, *y*−1, −*z*+1/2; (xi) *x*+1, *y*, *z*; (xii) −*x*+1, *y*, −*z*+1/2; (xiii) −*x*+1, *y*+1, −*z*+1/2.

### Hydrogen-bond geometry (Å, º)

**Table:**

*D*—H···*A*	*D*—H	H···*A*	*D*···*A*	*D*—H···*A*
N3_3—H3_3···O9_3	1.02 (1)	2.20 (1)	2.6086 (7)	102 (1)
N5_3—H5_3···O8_3^viii^	1.02 (1)	1.93 (1)	2.9070 (7)	162 (1)
N5_3—H5_3···O9_3^viii^	1.02 (1)	2.56 (1)	3.3977 (6)	140 (1)
N12_4—H12_4···O18_5	1.016 (5)	1.984 (8)	2.9628 (7)	160.9 (4)
N12_4—H12_4···O18_5^ii^	1.016 (5)	2.659 (9)	3.1515 (8)	110 (2)
N14_4—H14_4···O16_4^ix^	1.03 (1)	2.23 (1)	3.1553 (6)	150 (2)
N14_4—H14_4···O16_4^vii^	1.03 (1)	2.23 (1)	3.1553 (7)	150 (2)
O18_5—H18*B*_5···O16_4^xii^	0.96 (1)	2.58 (1)	3.3013 (8)	133 (2)
O18_5—H18*A*_5···N1_3^viii^	0.97 (1)	1.93 (1)	2.8927 (9)	173 (1)

Symmetry codes: (ii) −*x*+2, *y*, −*z*+1/2; (vii) −*x*+1, *y*−1, −*z*+1/2; (viii) *x*, *y*+1, *z*; (ix) *x*, *y*−1, *z*; (xii) −*x*+1, *y*, −*z*+1/2.
